# College and Hospital Notes

**Published:** 1900-01

**Authors:** 


### COLLEGE AND HOSPITAL NOTES.

The new City Hospital was formally opened at Owensboro, Ky., Thanksgiving Day, November 30, 1899. It was made an important event in that city, the dedication services having occupied the day and evening.

The opening service occurred at the buildings in the morning, where a committee received visitors from 9 to 11 a. m. and from 2 to 4 p. m. It was estimated that over 2,000 persons visited the buildings during the day.

The dedication services proper took place at Temple Theatre, beginning at 7.30 p. m. Every seat of the opera house was filled and many went who could not be seated. The services were opened with prayer by Rev. E. E. Smith, followed by an address on the History of the institution, by Mayor W. P. Small. Dr. C. H. Todd then introduced Dr. L. S. McMurtry, of Louisville, the chief speaker of the occasion, and who made the dedicatory address. It was a graceful speech and most appropriate to the occasion.

After the close of the services at the Temple Dr. McMurtry was entertained at luncheon at the Rudd House by Mayor Small, other city officials, hospital officers and citizens.

The buildings and grounds cost about $18,000. The grounds cost $7,000 and were presented to the city by the German School Association for hospital purposes, and the city and German School Association will jointly operate the hospital. Several hundred dollars were contributed toward equipping the buildings by some of the local fraternal societies. The main building is a handsome brick structure, and is one of the best equipped hospitals in the south. There are three rooms fitted up for patients who are able to pay and several wards for indigent patients. It has an elevator and the operating room is fitted up with all improved appliances.

---

A CHILDREN'S hospital has lately been built on the grounds of the Charity Hospital at New Orleans, which is one of the most complete hospitals of its kind in the country. It was projected, built, equipped and furnished by Mrs. Richard A. Milliken as a memorial to her husband and daughter. Mr. Milliken, the wealthy Louisiana sugar planter, received injuries about four years ago in a street railway accident from which he died, and Miss Milliken, aged 19 years, was drowned at Bar Harbor, Me., in the summer of 1888.

The hospital is four stories high, contains 175 beds, arranged in separate medical and surgical wards for boys and girls. It has surgical operating rooms, bath rooms, lavatories, and all accessories constructed according to the most approved plans as to plumbing, marble wainscoting, tiling and ventilation. Besides it has a kindergarten where convalescents and invalids can be entertained and instructed. Its total cost as it now stands was about $125,000 and it is a noble monument of philanthropic charity, admission to its wards being absolutely free.

---

SISTER M. FLORENCE, who for twenty-five years has had charge of the Sisters' of Charity Hospital, at Buffalo, has been transferred to the mother house at Emmittsburg, Md. Sister Cynerine, also with the same hospital for seventeen years, has been placed in charge of St. Mary's Hospital, at Detroit, Mich. Two Sisters from Emmittsburg take the places made vacant by the transfer of Sisters Florence and Cynerine.

---

IT IS a matter of considerable surprise that no provision for nursing students temporarily ill has been made heretofore in our colleges. Mrs. Edward Storrs Atwater, of Poughkeepsie, appreciating this need at Vassar, has given money for an infirmary. It is to be a memorial of her father, Charles W. Swift, for many years a trustee of the college. It is expected that only the foundations of the building will be laid this winter, but the infirmary will be completed and ready for occupancy next autumn.

Hon. D. S. Alexander, M. C., introduced in the House of Representatives, December 11, 1899, a bill providing for the proposed Marine Hospital at Buffalo.

The measure calls for an expenditure of $125,000. It was referred to the committee on public buildings and grounds and the plan is to submit it to the Secretary of the Treasury for his inspection and any suggestions he may have to offer. It authorises the Secretary of the Treasury to pursue precisely the same course in obtaining a site as is pursued in the case of a post office building. It is desirable that the hospital shall be located near the center of Buffalo.

